# Intermittent Supplementation with Far-Red Light Accelerates Leaf and Bud Development and Increases Yield in Lettuce

**DOI:** 10.3390/plants14010139

**Published:** 2025-01-06

**Authors:** Yanke Liu, Rong Ye, Xinying Gao, Rongcheng Lin, Yang Li

**Affiliations:** 1 Plant Factory R&D Center, Institute of Botany, Chinese Academy of Sciences, Beijing 100093, China; liuyanke22@mails.ucas.ac.cn (Y.L.); yerong23@mails.ucas.ac.cn (R.Y.); gaoxinying23@mails.ucas.ac.cn (X.G.); 2 University of Chinese Academy of Sciences, Beijing 100049, China; 3 Xianghu Laboratory, Hangzhou 311231, China; linrongcheng@xhlab.ac.cn; 4 China National Botanical Garden, Beijing 100093, China

**Keywords:** intermittent far-red light irradiation, light signaling, shade avoidance syndrome, lettuce, controlled environment agriculture

## Abstract

Supplementation with far-red light in controlled environment agriculture production can enhance yield by triggering the shade avoidance syndrome. However, the effectiveness of this yield enhancement can be further improved through intermittent far-red light supplementation. In this study, the effects are explored of varying far-red light photon intensities and intermittent exposure durations—specifically at 5, 15, 30, and 45 min intervals—on the growth and development of lettuce (*Lactuca sativa*) in plant factories, while maintaining a constant red light photon flux and daily light integral. The results showed that compared to constant far-red light, 30 min intermittent far-red light increased yield by 11.7% and the number of leaves and buds by 2.66. Furthermore, the various metrics demonstrated that intermittent far-red light supplementation enhanced the overall effectiveness of the far-red light treatment. This was validated by analyzing phytohormone content and the expression of genes related to hormone metabolism and transport at the tip of the lettuce stems. Transcriptome analysis revealed that the differences in gene expression between treatments were primarily concentrated in genes related to signaling, hormone metabolism, and transport. Weighted Gene Co-expression Network Analysis identified the co-expression modules associated with yield and quality. Additionally, dynamic expression analysis showed genes involved to far-red photoreception, response, and hormone metabolism and transport exhibited optimal rhythmic responses only under 30 min intermittent far-red light supplementation. This suggests that intermittent far-red light irradiation at 30 min intervals is the most effective for activating far-red light signaling influencing hormone metabolism and transport, thereby accelerating the growth of lettuce leaves and buds and ultimately increasing yield.

## 1. Introduction

Light serves a dual function in plants, providing the energy required for photosynthesis while also acting as a signal that regulates various plant responses. Photosynthetic pigments within the chloroplasts capture light energy for photosynthesis, whereas photoreceptors, which detect specific wavelengths of light, mediate the signaling processes [1].

Under natural light conditions, red and far-red light are primarily detected by a group of photoreceptors known as phytochromes, which include the five members of PhyA–PhyE. These phytochromes specifically sense light in the red and far-red spectrum, ranging from 600 to 750 nm. Structurally, phytochromes consist of the two main domains of the N-terminal photoreceptive region and the C-terminal regulatory region. This structure allows phytochromes to exist in two reversible states due to conformational changes in their chromophores. Under red light at 660 nm, phytochromes transition to the active Pfr state, while under far-red light at 730 nm, they revert to the inactive Pr state [2,3]. In their activated Pfr state, phytochromes interact with phytochrome-interacting factors (PIFs), resulting in the phosphorylation and subsequent degradation of PIFs, thereby regulating plant growth through hormone metabolism or other pathways [4,5]. The activation state of phytochromes is generally influenced by the ratio of red light to far-red light. The regulation of these pigments in response to an abundance of far-red light is known as the shade avoidance syndrome [6]. This phenomenon describes a plant’s ability to increase its access to light by elongating stems or petioles when exposed to a high proportion of far-red light, typically found in dense vegetation or shaded environments. While the shade avoidance syndrome often results in more rapid growth, it may also lead to a more slender plant morphology [7,8].

In plant factory production, effectively utilizing and balancing the two roles of light is fundamental for optimizing the light environment. In the previous research on suitable growing conditions for different crops, including sundews, tomatoes, bell peppers, green peppers, and lettuce, the most favorable light formulations all had red light as the main component [9,10,11,12,13,14]. Furthermore, the research has shown that augmenting red and blue light with other spectral components, such as green, violet, amber, far-red, and ultraviolet light, can positively affect specific crop parameters [15,16,17]. The identification of specific spectral compositions to enhance particular crop qualities remains a primary research goal in plant factory lighting.

Although the photosynthetic energy supply role of far-red light is limited, supplementing it with an appropriate amount of far-red light can enable its signaling function to induce the shade avoidance syndrome, thereby improving yields or promoting flowering. In lettuce production, substantial research indicates that the additional supplementation of far-red light across all spectral levels improves yield by increasing leaf number and total leaf area. The far-red light enhances lettuce’s ability to capture light while increasing total leaf area, thus contributing to higher yields [18,19,20]. However, overgrowth induced by far-red light supplementation often leads to a decline in certain nutritional indicators, possibly due to competition for resources during assimilation [21]. In addition, in the production of some other crops, supplemental far-red light can be used to promote tillering or flowering, or to induce fruit abortion, among other effects [22,23].

Notably, unlike the energetic action of light, the signaling response involves processes such as RNA transcription and protein translation, which have inherent response times [24]. This implies that under artificial plant lighting conditions, signaling light does not necessarily need to be consistently or continuously applied. Exploiting differences in the timing of irradiation may yield additional effects. Therefore, in this experiment, we maintained a constant total light dose while ensuring continuous red light irradiation and supplemented it with far-red light at varying intermittent intervals. This approach aimed to investigate whether temporal differences in far-red light supplementation are effective and to explore the underlying mechanisms.

## 2. Materials and Methods

### 2.1. Plant Materials and Growth Conditions

Green leaf lettuce (*Lactuca sativa* var. cv Rex) was used as the experimental material. The experiment was conducted in a fully enclosed laboratory. The LED lighting fixtures and growth modules utilized in the experiment were supplied by SANANBIO (Xiamen, China). Seeds were soaked in room temperature water for half an hour and sown in 2.5 cm × 2.5 cm × 2 cm sponge blocks, one per hole, and germinated in a germination box at a relative air humidity of 95% until radicle emergence. Seedlings were then grown under a white spectrum of 200 ± 10 µmol·m^−2^·s^−1^ for 12 h per day for approximately 10 days until they developed two true leaves. Seedlings were then transferred to hydroponic culture under the same light conditions (200 ± 10 µmol·m^−2^·s^−1^, 12 h/d), with the nutrient solution having an electrical conductivity (EC) of 1.2 dS/m and pH of 7.0, until they grew 4–5 true leaves in a week.

### 2.2. Experimental Light Design

After one week of growth under the above conditions, the lettuce was transplanted and cultivated under five different experimental light conditions, as shown in Table 1 and Supplementary Figure S1.

Uniformly sized seedlings were then selected and planted under one of the experimental lighting conditions, which involved 9 h of daily illumination, from 8:00 AM to 5:00 PM. The nutrient solution was maintained with an EC of 1.8 dS/m and a pH of 7.0, using the nutrient flow technique. The experimental area was kept at a temperature of 21 °C/19 °C (day/night), with a relative air humidity of 80%, a CO_2_ concentration of 400 ppm, which was monitored in real time by a monitor to ensure stability, and a planting row spacing of 15 cm × 20 cm. Each module contained 18 lettuce plants, and the photon flux density was measured using a PG200N Spectral PAR Meter (UPRtek, Taibei, China). The photon flux density of the LEDs was controlled based on measurements taken at the central axis of each module. All plants were grown within a 0.2 m radius from the central axis to minimize the effects of light bias. To ensure accuracy, each indicator in this experiment was assessed with three replicates, each containing 90 lettuce plants. The experiment was conducted four times to accommodate the harvest requirements of different treatment durations. After 7, 14, 21, and 28 days of treatment, the plants were harvested, and various parameters were measured. For phenotypic data, six plants per replicate were selected for measurement. For quality, metabolic, and gene expression analyses, tissue samples from standardized positions within the same module were pooled to ensure accuracy. The samples were immediately frozen in liquid nitrogen and stored at −80 °C until further analysis.

### 2.3. Determination of Lettuce Morphology

Lettuce was harvested at 7, 14, 21, and 28 days after the start of the experimental treatments by randomly selecting six samples to measure shoot fresh weight, number of leaves and buds, and stem length. The measurements were repeated three times to ensure data consistency and to plot the dynamics of the changes. On day 21 of the regular harvest, root weight, root length, and leaf morphology of the 10th and 18th leaves were measured in six randomly selected lettuce plants. The lengths and widths of leaves at fixed positions were measured to calculate the leaf index and used the portable leaf area meter YMJ-D (Topu yunnong, Hangzhou, China) to measure leaf area. The above-ground and below-ground parts of the lettuce were then separated, and their fresh weights were measured using an electronic balance (Wuxinhengqi, Hefei, China). They were then placed in an electric blast drying oven (Chuangxin, Beijing, China), at 105 °C for 20 min, and then dried at 80 °C for 2 days to a constant weight to measure the dry weights.

### 2.4. Determination of Photosynthetic Parameters and Photosynthetic Pigment Contents

After 21 days of treatment, the photosynthetic parameters were assessed using the LI-6800 PorTable Photosynthesis System (LI-COR, Lincoln, USA), the 10th to 12th leaves were taken using leaf chamber and read after the parameters had stabilized, including net photosynthesis rate (Pn), stomatal conductance (Gs), intercellular CO₂ concentration (Ci), among other parameters [25]. The light intensities used for these measurements corresponded to the experimental light intensities, with CO_2_ concentration at 400 ppm and relative humidity at 80%.

Photosynthetic pigment contents were determined following Wellburn’s method [26]. Approximately 0.2 g of fresh leaf tissue was immersed in 10 mL of a 1:1 (*v*/*v*) mixture of acetone and ethanol until the leaves became colorless, indicating complete extraction of the photosynthetic pigments. The absorbance of the extract was measured at 645 nm (OD_645_), 663 nm (OD_663_), and 470 nm (OD_470_) using a UV–visible spectrophotometer. The contents of the photosynthetic pigments were calculated as follows: chlorophyll a content (mg/g) = 12.7 × OD_663_ − 2.69 × OD_645_; chlorophyll b content (mg/g) = 22.9 × OD_645_ − 4.86 × OD_663_; carotenoid content (mg/g) = (1000 × OD_470_) − (3.27 × chlorophyll a) − (104 × chlorophyll b).

### 2.5. Determination of Lettuce Quality

The assessment of nutritional quality was conducted using leaves from fixed positions on different lettuce plants as samples. The leaves were crushed and homogenized before the soluble sugar content was measured using the anthrone method [27], where soluble sugars were reacted with anthrone reagent in acidic conditions, and the absorbance at 620 nm was measured to determine the sugar content. The soluble protein content was determined using the Coomassie brilliant blue method [28], proteins in the sample were bound by Coomassie brilliant blue dye, and the absorbance at 595 nm was measured to quantify the protein concentration. The vitamin C content was quantified via iodometric titration [29], ascorbic acid in the homogenized sample was titrated with iodine, and the absorbance at 520 nm (after the iodine titration) was used to calculate the vitamin C content based on the iodine consumption. The nitrate content was measured using the salicylic acid nitration method [30], nitrate ions in the sample reacted with salicylic acid, and the absorbance at 410 nm was measured to determine the nitrate concentration.

### 2.6. Quantitation of Hormone Levels of Lettuce Leaves and Petioles

For the quantitation of plant hormone levels, samples were systematically collected from lettuce stem tips. The analysis was conducted using the liquid chromatography–tandem mass spectrometry (LC-MS/MS) platform as previously described [31] provided by Metware Co., Ltd. (Wuhan, China) (www.metware.cn, accessed on 30 May 2024), which facilitated the detection of 108 different plant hormones and their secondary metabolites.

Briefly described below, the biological samples were ground into a fine powder using a grinder. Then, 10 μL of an internal standard mixture solution (100 ng/mL) and 1 mL of a methanol/water/formic acid (15:4:1, *v*/*v*/*v*) extraction solvent was added to the samples. After extraction, the samples were reconstituted in 80% methanol/water solution and filtered before being subjected to LC-MS/MS analysis. The data acquisition system included an ultra-performance liquid chromatography (UPLC) system (ExionLC™ AD) and a tandem mass spectrometry (MS/MS) system (QTRAP^®^ 6500+). The chromatographic conditions were as follows: the column used was a Waters ACQUITY UPLC HSS T3 C18 column (1.8 µm, 100 mm × 2.1 mm i.d.); the mobile phase A was ultra-pure water (containing 0.04% acetic acid), and mobile phase B was acetonitrile (containing 0.04% acetic acid). The gradient elution program was as follows: 0 min, A/B = 95:5 (*v*/*v*); 1.0 min, A/B = 95:5 (*v*/*v*); 8.0 min, A/B = 5:95 (*v*/*v*); 9.0 min, A/B = 5:95 (*v*/*v*); 9.1 min, A/B = 95:5 (*v*/*v*); 12.0 min, A/B = 95:5 (*v*/*v*). The flow rate was set to 0.35 mL/min, the column temperature was 40 °C, and the injection volume was 2 μL. The mass spectrometry data were then subjected to qualitative analysis based on a standard compound library constructed using the Metware Database (MWDB).

### 2.7. RNA-Sequencing Analysis

For RNA sequencing (RNA-Seq) analysis, leaf samples were collected from the fixed position of the 10th to 12th leaves of treatments on the 21st day of treatment. The samples were processed and analyzed by Sangon Biotech Co., Ltd. (Shanghai, China) (www.sangon.com, accessed on 30 April 2024). In brief, after RNA extraction, the RNA library was sequenced on the Illumina Platform, and HISAT2 was used to grade the quality of reads and compared with the reference genome. StringTie was performed to analyze the expression level, and DESeq2 was used to analyze the differentially expressed genes (DEGs), in which |log2FoldChange| ≥ 2 and j < 0.05 were considered significant. The Kyoto Encyclopedia of Genes and Genomes (KEGG) database, eukaryotic Orthologous Groups (KOG) database, GOATOOLS, and R language (www.majorbio.com, accessed on April 2024) were used to determine the main biological functions of DEGs enriched in KOG and KEGG enrichment analyses.

### 2.8. WGCNA Network Analysis

After filtering out genes with low expression levels from the RNA-seq results across the five experimental conditions, the FPKM data of the top 10% most variable DEGs were used for Weighted Gene Co-expression Network Analysis (WGCNA) using the R package “WGCNA”. An adjacency matrix was constructed with a soft threshold power of 9. Network interconnectedness was assessed by calculating the topological overlap using the “TOMdist” function with an unsigned TOM-type. Genes were grouped based on their connection strengths using average hierarchical clustering with the “hclust” function, according to the topological overlap dissimilarity measure (1-TOM). The resulting heatmap plot of topological overlap within the gene network visually represents the relationships among gene clusters [32]. To link physiological measurements with gene modules, the module eigengenes were correlated with lettuce yield and quality indicators. Correlations were performed for each physiological trait separately using the mean values, allowing us to associate the observed patterns between physiological traits and module eigengenes.

### 2.9. RT-qPCR Experiment

For quantitative PCR (qPCR) experiments, stem tips from the five experimental lighting conditions were taken at noon on the 21st day. To determine changes in gene expression over time within a treatment, leaf samples were collected at 14:00, 14:07, 14:15, 14:22, 14:30, 14:45, 15:00, 15:15, and 15:30 for FRC treatment group; at 14:00, 14:07, 14:15, 14:22, and 14:30 for FR15 treatment group; at 14:00, 14:15, 14:30, 14:45, and 15:00 for FR30 treatment group; and at 14:00, 14:22, 14:45, 15:07, and 15:30 for FR45 on the 21st day of treatment. These samples were immediately frozen in liquid nitrogen and stored at −80 °C. Total RNA was extracted using the RNA Easy Fast Plant Tissue Kit (Tiangen, Beijing, China), with quality assessment performed through agarose gel electrophoresis and analysis with a K2800 nucleic acid analyzer (Kaiao, Beijing, China). Subsequently, the RNA was reverse-transcribed into cDNA using Script III All-in-one RT mix with dsDNase kits (Huaxingbio, Shenyang, China). Fluorescent qPCR experiments were conducted using SYBR Green qPCR mix (Huaxingbio, Shenyang, China) and Light Cycler^®^ 480 II (Roche, Switzerland). The genes analyzed were selected based on RNA-Seq results, with gene sequences obtained from NCBI and primers designed using Primer Premier 5, detailed in Table S1. Each RT-qPCR experiment was performed with three biological replicates and three technical replicates per biological replicate. Ct values were determined using default settings, and relative gene expression levels were calculated using the 2^−ΔΔCt^ method [33]. The average Ct value of two housekeeping genes served as the internal reference for normalization.

### 2.10. Statistical Analyses

Experimental data were processed and visualized using SPSS 26, GraphPad Prism 5, and R studio 4.3.3 (2024. 09. 0 + 375) software. Statistical analysis involved one-way ANOVAs at a significance level of *p* < 0.05, employing Duncan’s multiple range test for post hoc comparisons. Correlation analysis and principal component analysis (PCA) were utilized to evaluate the relationships among various indicators of lettuce under different light treatments and their impact on lettuce traits.

## 3. Results

### 3.1. Intermittent Supplemental Irradiation Enhances the Effect of Far-Red Light on Lettuce Growth and Development

**Figure 1:**
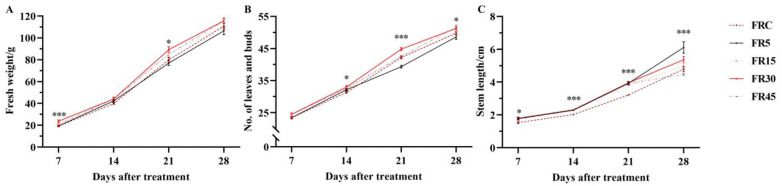
Dynamics of lettuce morphology at different days after treatment (DAT) harvests. Shoot fresh weight (**A**), number of leaves and buds (**B**), and stem length (**C**) of lettuce at different DAT. The values are the mean ± SEM of three replicates, * indicates significance results of one-way ANOVA, * *p* < 0.05 and *** *p* < 0.001.

Intermittent supplemental light enhanced the effect of far-red light on lettuce, even with the same total FR light exposure. The greatest difference in fresh weight was observed 21 days after treatment (DAT), with the FR30 treatment yielding 11.7% more lettuce compared to the FRC and 16% more compared to the FR5. Regarding the number of leaves and buds, at 21 DAT, the FR30 treatment also produced 2.66 more leaves and buds than the FRC treatment, and 5.5 more than the FR5 treatment. This increase in leaves and buds was not offset by the narrowing of fresh weight differences observed at 28 DAT. Additionally, among all of the DAT, lettuce subjected to intermittent far-red light treatments exhibited longer stems compared to those under continuous FRC treatment. Notably, the FR5 treatment resulted in significantly more slender stems than other treatments, with a markedly greater stem elongation observed at 28 DAT (Figure 1, Supplementary Figure S2 and Supplementary Table S2). Furthermore, shoot dry matter content (DMC) and root length were significantly higher under the FR30 treatment compared to other treatments. Although the root weight did not differ significantly among the five treatments, a significant reduction in root mass fraction was observed in the FR15 treatment (Supplementary Table S3).

**Figure 2:**
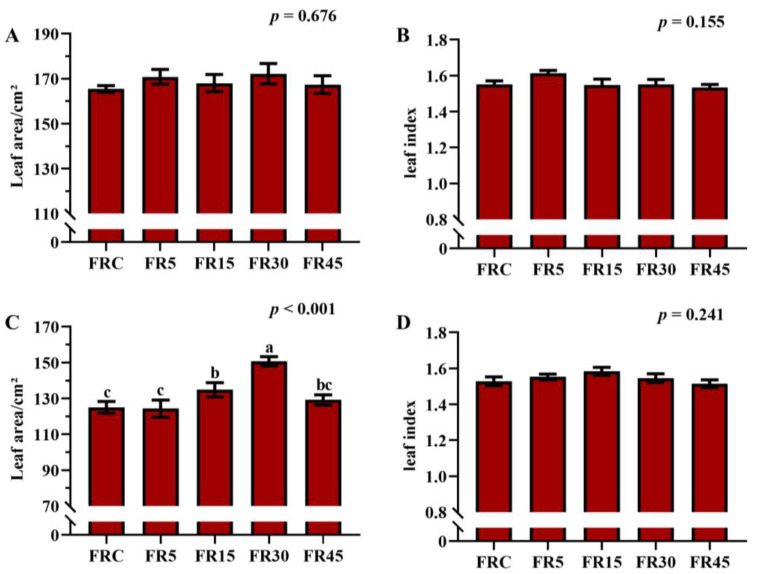
Leaf area and leaf index of lettuce under different treatments. Leaf area (**A**) and leaf index (**B**) of the 10th leaf. Leaf area (**C**) and leaf index (**D**) of the 18th leaf. The values are the mean ± SEM of three replicates, and the letters indicate the significant differences among different treatments (*p* < 0.05), significant results of one-way ANOVA are shown.

Leaf area of the 10th leaf of lettuce did not show significant differences between treatments, but the leaf area of the 18th leaf under the FR30 treatment was significantly larger than that of other treatments, with the smallest areas observed in the FRC and FR5 treatment. This is consistent with the differences in fresh weight and the number of leaves and buds at 21 DAT (Figure 2A,B and Supplementary Table S2). Moreover, there was no significant difference in the leaf index between the 10th and 18th leaves, suggesting that compared to continuous far-red light irradiation, intermittent far-red light supplementation accelerated the development of lettuce leaves and buds, causing earlier growth of leaves at the same positions (Figure 2C,D). Thus, intermittent irradiation with far-red light enhances the yield-promoting effects of far-red light supplementation on leaf and bud development, even with the same total amount of far-red light.

### 3.2. Overgrowth Caused by Intermittent Supplemental Light Reduces Assimilation of Nutrients

Intermittent supplemental far-red light irradiation decreased photosynthetic pigment levels while increasing fresh weight and leaf number. The FR30 treatment, which had the most significant yield increase, exhibited significantly lower levels of photosynthetic pigments compared to the other treatments (Table 2). However, the net photosynthetic rate (Pn) did not show significant differences among treatments, likely due to the increase in stomatal conductance and intercellular carbon dioxide concentration under the intermittent supplemental treatments (Supplementary Table S3).

**Figure 3:**
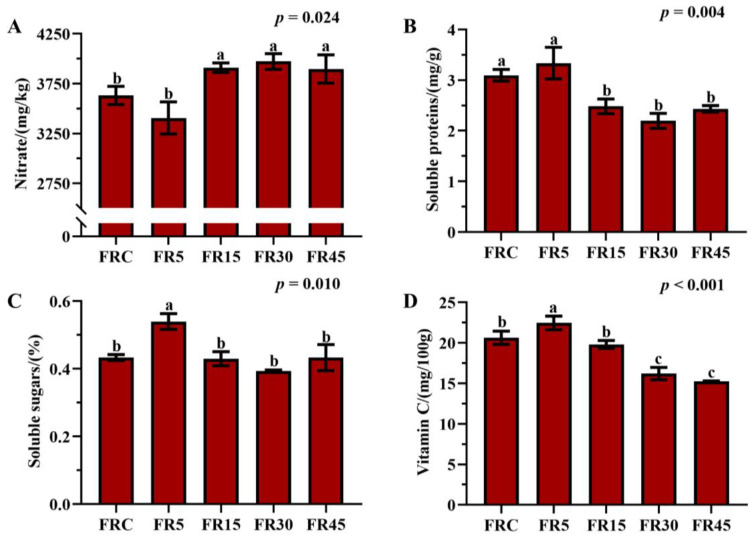
Differences in lettuce quality under different treatments. Nitrate (**A**), soluble proteins (**B**), soluble sugars (**C**), and vitamin C (**D**). The values are the mean ± SEM of three replicates, and the letters indicate the significant differences among different treatments (*p* < 0.05); significant results of one-way ANOVA are shown.

Nitrate content in lettuce was highest in the FR30 treatment but significantly lower in both the FRC and FR5 treatments. Conversely, soluble sugars, soluble proteins, and vitamin C were highest in the FR5 treatment and significantly lower in the FR30 treatment (Figure 3). The rapid growth associated with intermittent exposure to far-red light may have hindered the assimilation of inorganic matter absorbed by the lettuce, resulting in a reduction in the content of several key nutrients, including photosynthetic pigments. This aligns with the production pattern where more mature plants typically exhibit lower nutritional quality.

### 3.3. Hormone Accumulation at the Lettuce Stem Tip Correlates Strongly with Leaf Bud Development and Stem Length

**Figure 4:**
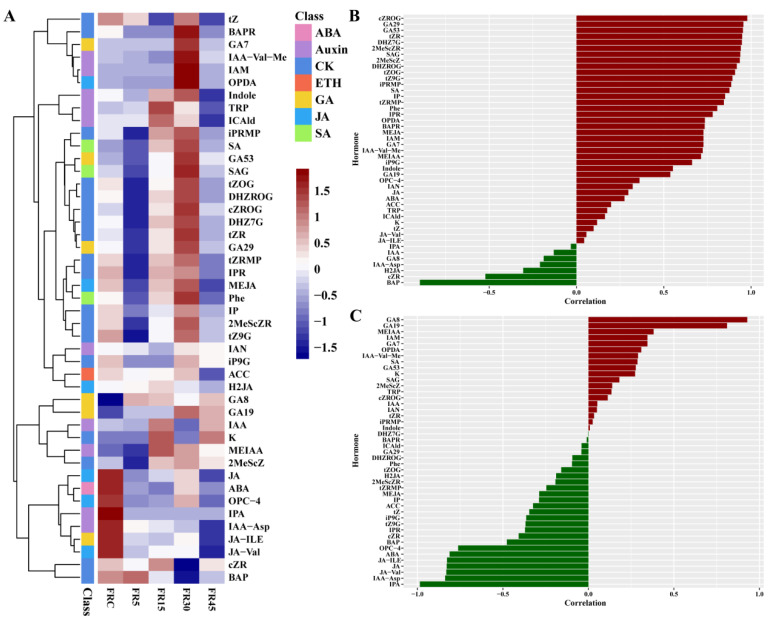
Effects of intermittent supplementation with far-red light on hormone levels in lettuce. (**A**) Heat map of hormone content of lettuce stem tips under different treatments. Data were standardized by unit variance scaling (UV), and the means of three replicates are shown. (**B**) Correlation of different hormones with number of leaves and buds. (**C**) Correlation of different hormones with stem length.

Intermittent supplemental far-red light increased lettuce yield and induced stem elongation by accelerating leaf bud development. Lettuce grows by producing buds through the apical meristem at the stem apex, which subsequently develop into leaves. This process is driven by the accumulation of cytokinins [34]. Consequently, we measured hormone levels at the stem tip where buds emerge. The results showed that most cytokinins were highest in the FR30 treatment, which had the greatest number of leaves and buds, and lowest in the FR5 treatment, which had the fewest. In addition to cytokinins, three salicylic acids (SA, SAG, and Phe) and two gibberellins (GA53 and GA29) exhibited similar distribution. Meanwhile, the levels of jasmonic acid and abscisic acid (ABA) were significantly higher in the FRC treatment compared to the other treatments (Figure 4A).

Correlation analysis between the hormone content at the stem tip and the number of leaves, buds, and stem length of lettuce demonstrated that all 16 phytohormones, including Phe and tZRMP, were strongly positively correlated with the number of leaves and buds. Most of these phytohormones were cytokinins, salicylic acids, and two types of gibberellins. Interestingly, one cytokinin, BAP, showed a significant negative correlation with the number of leaves and buds (Figure 4B). Further correlation analysis of stem length demonstrated that the content of two gibberellins (GA8 and GA19) at the stem tip was highly positively correlated with stem length, whereas ABA, three jasmonic acids, and two auxins (IPA and IAA-Asp) were highly negatively correlated with stem length (Figure 4C).

### 3.4. Expression of Genes Related to Hormone Metabolism and Transport in Lettuce Stem Tips

Expression analysis was performed on several hormone metabolism and transport-related genes in the stem tip, including the cytokinin metabolism genes *Lactuca sativa LONELY GUY 5 (LsLOG5)* and *Lactuca sativa ISOPENTENYLTRANSFERASE 3 (LsIPT3*), the gibberellin metabolism-related gene *Lactuca sativa GIBBERELLIN 20-OXIDASE 2* (*LsGA20OX2*), the abscisic acid metabolism gene *Lactuca sativa 9-CIS-EPOXYCAROTENOID DIOXYGENASE 2 (LsNCED2*), the auxin metabolism gene *Lactuca sativa PROBABLE INDOLE-3-PYRUVATE MONOOXYGENASE YUCCA 5* (*LsYUC5*), the auxin response gene *Lactuca sativa SMALL AUXIN UP RNA 71 (LsSAUR71*), the auxin inactivation and degradation gene *Lactuca sativa GRETCHEN HAGEN 3.6 (LsGH3.6*), and the phytohormone transport-related gene *Lactuca sativa ATP-BINDING CASSETTE B6 (LsABCB6*).

**Figure 5:**
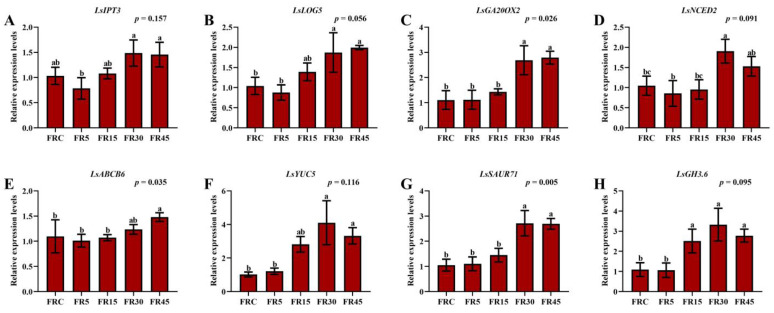
Expression of genes related to hormone metabolism and transportation in lettuce stem tips under different treatments. (**A**) Relative expression levels of *LsIPT3*. (**B**) Relative expression levels of *LsLOG3*. (**C**) Relative expression levels of *LsGA20OX2*. (**D**) Relative expression levels of *LsNCED2*. (**E**) Relative expression levels of *LsABCB6*. (**F**) Relative expression levels of *LsYUC5*. (**G**) Relative expression levels of *LsSAUR71*. (**H**) Relative expression levels of *LsGH3.6*. The values are the mean ± SEM of three replicates, and the letters indicate the significant differences among different treatments (*p* < 0.05), significant results of one-way ANOVA are shown.

The results showed that almost all genes exhibited the lowest expression levels in the FR5 treatment, with similarly low expression in the FRC treatment. In contrast, most genes were most highly expressed in the FR30 treatment, with similarly high expression levels in the FR45 treatment (Figure 5). Interestingly, some genes with entirely opposite roles, such as *LsYUC5*, *LsSAUR71*, and *LsGH3.6*, exhibited similar expression profiles (Figure 5F–H). The simultaneous high expression of these three genes suggests that auxin at the stem tip of lettuce is undergoing a process of high synthesis, strong response, and rapid deactivation and degradation under the FR30 treatment. In addition, differential expression of the plant hormone transport-related gene *LsABCB6* was observed (Figure 5E), indicating the complex regulatory role of hormone transport, which may explain the lower hormone accumulation in the stem tips of the FR45 treatment.

These findings imply that hormone metabolism at the stem tip is in a state of complex equilibrium, leading to distinct content profiles. Notably, although the magnitude of changes in gene expression varied greatly, the overall trend exhibited a striking similarity. Given that the stem tip of lettuce is not the primary site for sensing far-red light irradiation, due to the shading effect of the leaves and the variability in plant tissue size and structure, the observed expression patterns may be influenced by cross-tissue communication of hormones or other signaling molecules from tissues directly exposed to substantial amounts of far-red light.

### 3.5. Transcriptome Analysis Reveals That Intermittent Supplemental Irradiation with Far-Red Light Primarily Induces Signaling-Related Differences

**Figure 6:**
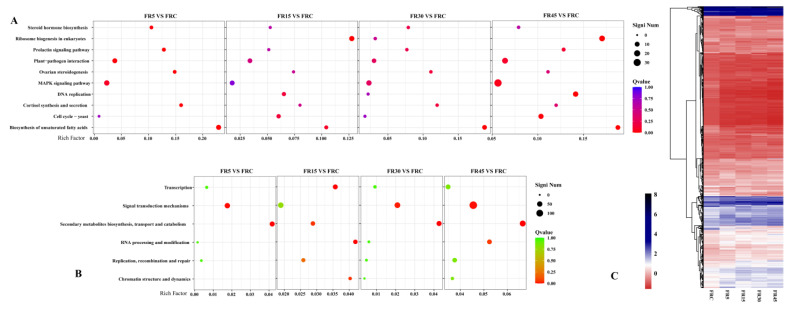
Effects of intermittent supplementation with far-red light on the lettuce transcriptome. (**A**) KEGG enrichment plots for significantly differentially expressed genes (DEGs). (**B**) KOG enrichment plots for significantly DEGs. Rich factor refers to the ratio of the number of DEGs located under this term to the total number of genes located in this pathway among all annotated genes. The larger the rich factor, the greater the enrichment. (**C**) Heat map of all significantly DEGs between treatments, with expression values represented as mean log2 (TPM+1) of three replicates.

In the transcriptome analysis under different treatments, pairwise comparisons identified a total of 1642 significant differentially expressed genes (DEGs). The expression of most DEGs showed less variation among the intermittent supplemental far-red light treatments, with more pronounced differences observed between these treatments and the FRC treatment (Figure 6C).

KEGG pathway enrichment analysis of DEGs from the four intermittent far-red light treatments compared to the FRC treatment revealed that these intermittent treatments primarily affected pathways such as the MAPK signaling pathway, biosynthesis of unsaturated fatty acids, steroid hormone biosynthesis, and other pathways directly or indirectly involved in plant signaling, hormone metabolism, or transport (Figure 6A). Meanwhile, KOG enrichment analysis also indicated that intermittent treatment, compared to FRC treatment, led to enrichment in functions related to signal transduction mechanisms and the biosynthesis, transport, and catabolism of secondary metabolites (Figure 6B). These results highlight the differential response in signal transduction and metabolic transport of plant signaling molecules under intermittent far-red light treatment compared to FRC treatment.

**Figure 7:**
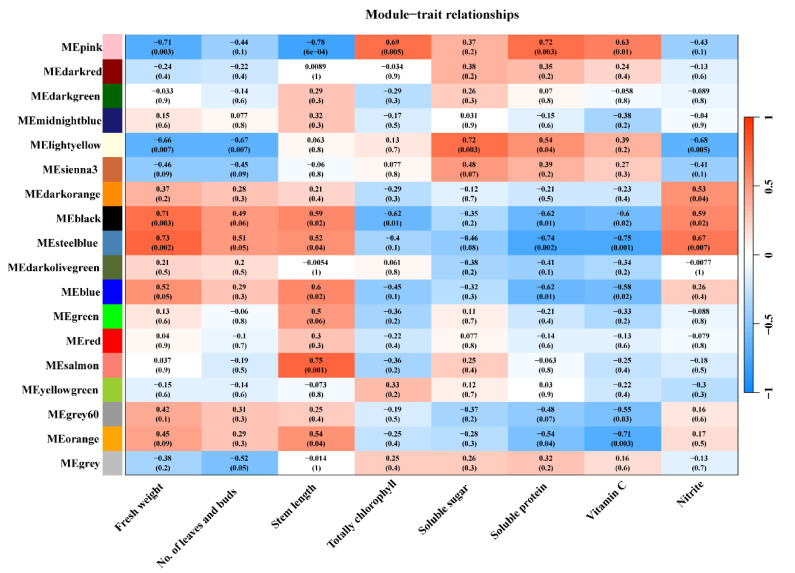
Co-expressed modules in relation to lettuce yield and quality indicators. Numbers in the heatmap denote the correlations of the corresponding module eigengenes and traits, and the values in the brackets indicate the *p*-value for their correlations.

WGCNA was performed on the top 10% most variable DEGs, identifying a total of 18 co-expressed gene modules after merging, with module sizes ranging from 36 to 1625 genes (Supplementary Figure S3). Correlation analysis between these gene modules and key lettuce yield and quality indicators revealed that the genes in the MEblack, MEsteelblue, and MEpink modules were most strongly associated with yield. Additionally, certain modules showed higher correlations with other traits (Figure 7). Detailed information on the genes within each module is provided in the Supplemental Data Set S3.

### 3.6. Dynamic Expression of Genes Related to Far-Red Light Response and Hormone Metabolism and Transport

**Figure 8:**
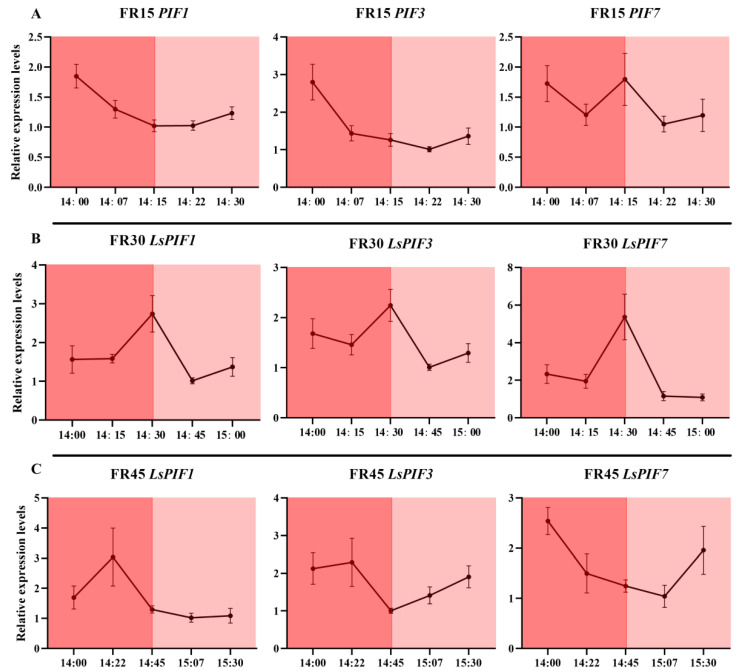
Dynamic expression of three phytochrome-responsive genes *LsPIF1*, *LsPIF3*, and *LsPIF7* under different intermittent far-red light treatments. (**A**) Dynamic expression in the FR15 treatment, with far-red light on from 14:00 to 14:15 and off from 14:15 to 14:30. (**B**) Dynamic expression in the FR30 treatment, with far-red light on from 14:00 to 14:30 and off from 14:30 to 15:00. (**C**) Dynamic expression in the FR45 treatment, with far-red light on from 14:00 to 14:45 and off from 14:45 to 15:30. The values are the mean ± SEM of three replicates.

We measured the dynamic gene expression of *Lactuca sativa PHYTOCHROME INTERACTING FACTOR 1 (LsPIF1), Lactuca sativa PHYTOCHROME INTERACTING FACTOR 3 (LsPIF3)*, and *Lactuca sativa PHYTOCHROME INTERACTING FACTOR 7 (LsPIF7)*, which are directly affected by conformational changes in phytochromes, at different times throughout the replenishment cycle of the intermittent irradiation treatment. The results showed that 15 min of far-red light irradiation was insufficient to induce a substantial response in *LsPIF1* and *LsPIF3* under the FR15 treatment, with only *LsPIF7* exhibiting an elevated expression (Figure 8A). In contrast, under the FR30 treatment, where far-red light was continuously irradiated for 30 min, all three *LsPIFs* exhibited a significant response and reached their highest expression levels at 14:30 (Figure 8B). However, in the FR45 treatment, the 45 min far-red light irradiation exceeded the required response time for the *LsPIFs*, resulting in a lack of sustained high expression at the 14:45 time point, despite the continued far-red light irradiation (Figure 8C).

**Figure 9:**
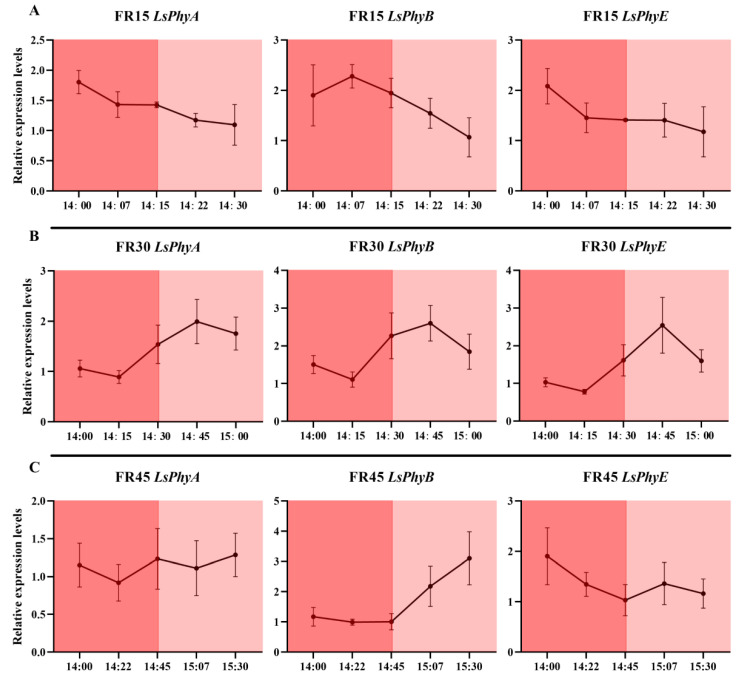
Dynamic expression of three phytochrome genes *LsPhyA*, *LsPhyB*, and *LsPhyE* under different intermittent far-red light treatments. (**A**) Dynamic expression in the FR15 treatment, with far-red light on from 14:00 to 14:15 and off from 14:15 to 14:30. (**B**) Dynamic expression in the FR30 treatment, with far-red light on from 14:00 to 14:30 and off from 14:30 to 15:00. (**C**) Dynamic expression in the FR45 treatment, with far-red light on from 14:00 to 14:45 and off from 14:45 to 15:30. The values are the mean ± SEM of three replicates.

Regarding the expression of the phytochrome genes themselves, the three phytochrome genes, *LsPhyA*, *LsPhyB,* and *LsPhyE*, similarly failed to respond promptly under the 15 min far-red light irradiation in the FR15 treatment (Figure 9A). However, their highest expression levels were observed with the 30 min far-red light irradiation in the FR30 treatment at 14:45 (Figure 9B). This suggests that the expression of phytochrome genes may lag behind the response of *LsPIFs*, requiring additional time to fully respond and alter gene expression. In the FR45 treatment, the extended 45 min irradiation may lead to variable signaling times, resulting in the inconsistent expression of the three phytochrome genes (Figure 9C).

**Figure 10:**
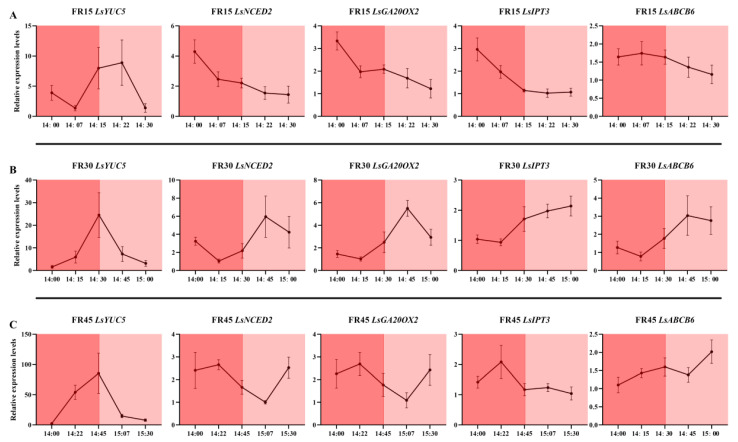
Dynamic expression of five hormone metabolism or transport-related genes *LsYUC5*, *LsNCED2*, *LsGA20OX2*, *LsIPT3*, and *LsABCB6* under different intermittent far-red light treatments. (**A**) Dynamic expression in the FR15 treatment, with far-red light on from 14:00 to 14:15 and off from 14:15 to 14:30. (**B**) Dynamic expression in the FR30 treatment, with far-red light on from 14:00 to 14:30 and off from 14:30 to 15:00. (**C**) Dynamic expression in the FR45 treatment, with far-red light on from 14:00 to 14:45 and off from 14:45 to 15:30. The values are the mean ± SEM of three replicates.

In the dynamic expression analysis of genes related to hormone metabolism and transport, it was found that all genes, except *LsYUC5*, failed to respond to the 15 min far-red light irradiation in the FR15 treatment (Figure 10A). In the FR30 treatment, which involved 30 min of far-red light, *LsYUC5* exhibited a rapid and robust response, while the other genes displayed a response pattern consistent with that of the phytochrome genes, peaking at 14:45. In the FR45 treatment with 45 min of far-red light, *LsYUC5* continued to respond rapidly and strongly (Figure 10B); however, the responses of the other genes were inconsistent and disorganized (Figure 10C).

We also examined the gene expression at corresponding time points within 90 min under the FRC treatment. The results showed that under continuous but low-intensity far-red light exposure, the genes mentioned exhibited rhythmic expression between approximately 20 and 40 min. However, the overall amplitude was lower compared to the intermittent far-red light supplementation treatment, and the rhythmic timing points varied between different genes. Under the FRC environment, *LsPIFs* reached their peak expression again 30-45 min after the lowest point, but the overall amplitude was only 1.5 to 2 times higher than the minimum, which was lower than the 2.5 to 5 times increase observed under the intermittent light treatment (Supplementary Figure S4). The same pattern was observed in the photoreceptor and hormone metabolism-/transport-related genes. The time intervals from the lowest to the highest expression points ranged from 20 to 45 min, but the overall amplitude was clearly lower than that in the intermittent far-red light supplementation treatment. Among these genes, *LsYUC5*, which responds rapidly to intermittent light supplementation, also showed rapid changes in the FRC treatment, but the overall amplitude was still weak (Supplementary Figures S5 and S6).

### 3.7. Principal Component Analysis

**Figure 11:**
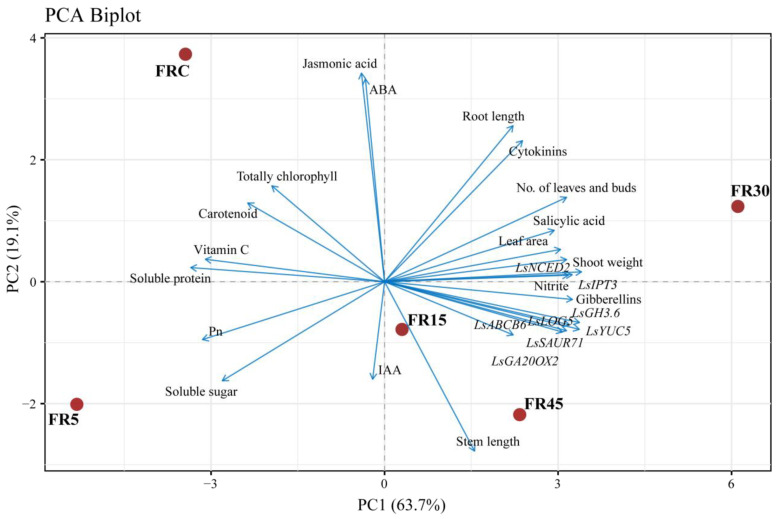
Principal component analysis (PCA) showing differences and correlations between morphology, metabolism, and transcription in lettuce.

Principal component analysis (PCA) indicated that the FR30 treatment was the furthest from the FR5 treatment, which aligned with the greatest differences observed between the two treatments across all indices. Nutrient indices, including vitamin C, soluble protein, soluble sugar, and photosynthetic pigments, were highest in the FR5 treatment, demonstrating a comparative correlation among these indices. Conversely, indicators crucial for yield, such as fresh weight, number of leaves and buds, cytokinins, and gibberellins, pointed to the FR30 treatment, highlighting its superiority in yield (Figure 11).

## 4. Discussion

### 4.1. Intermittent Supplementation of Far-Red Light Enhances the Effect of Constant Far-Red Light Supplementation

Excessive far-red light can cause plants to perceive themselves as being in a low-light environment, triggering a shade avoidance response that causes the stem elongation and increased leaf area to optimize light capture [35]. Research in controlled environment agriculture has shown that supplemental far-red light encourages lettuce to produce more leaves, thereby increasing the total leaf area and the leaf area available that can be spread out over the planting density. This enhanced light capture ultimately leads to increased yields [18,19,36]. In most studies investigating the effects of far-red light irradiation on bud growth, increased far-red light has been observed to promote earlier bud emergence [37,38]. However, the total number of buds often decreases [39]. Additionally, increased far-red light also promotes stem elongation. The relationship between bud outgrowth and stem growth under far-red light is believed to result from the combined regulation of multiple plant hormones, including auxins, gibberellins, and cytokinins [40,41,42]. Far-red light also promotes stem elongation, with the relationship between bud outgrowth and stem growth under far-red light believed to result from the combined regulation of multiple plant hormones, including auxins, gibberellins, and cytokinins [43,44]. However, excessive far-red light can also lead to overgrowth and a decline in some key nutrients, such as photosynthetic pigments and soluble proteins, due to increased assimilative competition [21,45].

Here, our research shows that intermittent far-red irradiation, when maintaining the same total amount of light, enhances its effectiveness. Compared to the FRC treatment with no constant far-red light exposure, the FR30 treatment, which involved intermittent far-red light, resulted in an 11.7% increase in yield at 21 DAT and an increase of 2.66 leaves and buds. However, this yield-enhancing effect diminished by 28 DAT (Figure 1 and Supplementary Table S2). The area of the 18th leaf increased significantly, while that of the 10th leaf remained unchanged. The increased number of leaves and buds caused the leaves at the same positions to approach full maturity more quickly (Figure 2). Considering previous studies, the increase in lettuce yield under far-red light supplementation may result from an accelerated growth cycle. Intermittent far-red light supplementation amplifies this effect by expediting leaf emergence. The larger leaf area allows for enhanced light capture, sustaining this advantage during the vegetative growth phase. However, as the vegetative growth phase nears completion, this growth cycle advantage diminishes, leading to the observed results.

Additionally, the stem elongation and subsequent analysis of phytohormone content at the stem tip revealed that increased cytokinins enhanced bud and leaf development by accelerating bud division and formation (Figure 4). However, this also meant that the competition between yield and quality in lettuce induced by far-red light intensified, with nearly all quality indicators, including photosynthetic pigment content, declining as the yield increased (Table 2, Figure 3). This decline continues to affect the photosynthetic rate, which may be one of the reasons why the differences between treatments begin to diminish after 28 DAT (Figure 1 and Supplementary Tables S2 and S4). However, this underscores the effectiveness of intermittent far-red light supplementation as a superior method overall, achieving more dramatic results with a smaller amount of far-red light.

### 4.2. Differential Response of Signals Under Far-Red Light Irradiation with Varying Interval Times

Far-red light perception in plants is primarily mediated by phytochromes, which exist in the following two interconvertible states: the active Pfr state induced by red light at 660 nm and the inactive Pr state induced by far-red light at 730 nm [7]. Both red and far-red light can influence phytohormone metabolism and transport through downstream signaling pathways initiated by phytochrome conformational changes. Far-red light exerts its effects by interacting with PIFs, which regulate the expression of genes involved in plant hormone processes, such as *YUC*, *GA20*, and *NCED* [46,47]. These processes play a crucial role in shaping plant growth and development [48,49]. Phytochromes and their complex downstream signaling mechanisms require time to respond, with response times varying depending on the specific signal [24,50].

To gain a comprehensive understanding of the dynamic gene expression influenced by the supplemental light cycle, we began our gene expression verification at 14:00 on the 21st day of treatment. By this time, the genes had been exposed to six hours of light, allowing us to observe the residual effects and sustained expression patterns resulting from the previous six-hour light cycle. This approach facilitates a robust analysis of the temporal dynamics under the given photoperiodic conditions. In our study, it was found that the FR30 treatment with 30 min intervals of irradiation resulted in strong rhythmicity for nearly all types of genes, suggesting that far-red light signaling elicited an adequate and appropriate response. In contrast, the FR15 treatment with 15 min intermittent irradiation was unable to respond in a timely manner, while the FR45 treatment lacked good rhythm and wasted replenishment time due to the long intervals between cycles (Figure 8, Figure 9 and Figure 10). In our analysis of the dynamic expression levels of the aforementioned genes under FRC treatment, nearly all genes exhibited rhythmic upregulation with intervals of 20–40 min, albeit with much smaller amplitudes compared to the intermittent light treatments (Supplementary Figures S4–S6). This indicates that even under continuous far-red light irradiation, the plant’s response to far-red light remains periodic. Continuous exposure does not sustain gene activation but instead triggers responses at defined intervals, suggesting that far-red light irradiation beyond the plant’s response window constitutes an inefficient use of energy. Intermittent far-red light supplementation at specific intervals can elicit stronger responses with the same dosage. Among the treatments evaluated, the FR30 treatment demonstrated the most pronounced effects of far-red light, highlighting its effectiveness as an optimized timing strategy.

It is noteworthy that the auxin metabolism gene *LsYUC5* responds strongly to far-red light at a very rapid rate, possibly comparable to the rate of the *LsPIF7* gene, which interacts directly with the phytochrome. This indicates a well-regulated, normal expression regardless of the far-red light treatment interval. Auxins are known to promote stem elongation, and in this experiment, all intermittent far-red light treatments resulted in stem lengths exceeding those of the FRC treatment, with consistent results in the first 21 DAT. This effect may be linked to the rapid response of the *LsYUC5* gene [51]. Interestingly, while most intermittent far-red light treatments triggered a complete shade avoidance syndrome, the FR5 treatment only partially engaged the auxin aspect of this response, affecting stem length without increasing fresh weight (Figure 1 and Supplementary Table S2).

### 4.3. Hypothesis for an Artificial Lighting Model That Fully Leverages Both the Signaling and Energetic Effects of Light on Plant

Much work has been performed to fully understand the implications of artificial light for plants, including efforts to isolate the monochromatic spectrum from the natural full spectrum to explore its role, and subsequent research combining these spectra for the best effects [10,11,12,52]. Additionally, considerable research has focused on specific light patterns of discontinuous illumination [53,54,55]. However, all of these research efforts should be based on the principle of the dual role of light in energy supply and signaling response for plant growth. While continuous illumination is essential for energy supply, we demonstrate here that non-constant intermittent illumination enhances signaling efficiency by leveraging the time-varying nature of signaling.

The signaling action of far-red light requires phytochromes to be phosphorylated before activating their complex molecular mechanisms; in contrast, cryptochromes, which sense blue light, act by altering their protein interaction affinities upon blue light exposure, and this suggests that different light signals may have varying response times due to their distinct modes of activation [56,57,58]. In our previous studies on intermittent supplemental blue light, we found that intermittent blue light treatments accelerated the emergence of the leafy head trait in lettuce. Compared to continuous light conditions, intermittent blue light treatment with 15 min intervals reduced the number of days required for half of the lettuce plants to reach the heading stage by eight days. This suggests that blue and far-red light induce signaling responses at different speeds and amplitudes, leading to varied developmental outcomes.

A well-established artificial lighting environment for plants should provide a continuous high level of energetic light while periodically adjusting a portion of the light spectrum to induce various signaling responses. This approach aims to optimize both energy utilization and signaling effects. Consequently, several questions arise, as follows: What types of light have signaling effects? What are the specific signaling effects of light? How long does it take for each signal to elicit its response? How long do these signaling responses last before supplemental irradiation is required? What is the optimal dose of light to elicit a signaling response, and is a constant dose necessary? Once we answer these questions, how do we design and calculate such a sophisticated lighting system? Clearly, there is still much to be explored in the field of artificial light for plants.

## 5. Conclusions

While maintaining the same total light exposure, intermittent far-red light supplementation is more effective than constant far-red light in accelerating the development of additional lettuce leaves and buds, thereby increasing yield. This is because far-red light signaling requires time, and when delivered at optimal intervals, it triggers a stronger signaling response, influencing hormone metabolism and transport. Consequently, this leads to hormone accumulation at the stem tip, accelerating the growth of leaves and buds.

## Figures and Tables

**Table 1:** Experimental lighting conditions.

Treatment Code	Red LEDs Photon Flux Density (µmol·m^−2^·s^−1^)	Far-Red LEDs Photon Flux Density (µmol·m^−2^·s^−1^)	Supplemental Frequency	Red/Far-Red Ratio (on, off)	PSS Value (on, off)
FRC	180 ± 10	30 ± 2	Constant irradiation	6	0.84
FR5	180 ± 10	60 ± 5	5 min on, 5 min off	3, N/A	0.82, 0.88
FR15	180 ± 10	60 ± 5	15 min on, 15 min off	3, N/A	0.82, 0.88
FR30	180 ± 10	60 ± 5	30 min on, 30 min off	3, N/A	0.82, 0.88
FR45	180 ± 10	60 ± 5	45 min on, 45 min off	3, N/A	0.82, 0.88

PSS value, phytochrome photostationary state value.

**Table 2:** Photosynthetic pigment contents of lettuce under different treatments.

Treatment Code	Chlorophyll a (mg/g)	Chlorophyll b (mg/g)	Chlorophyll a:b	Total Chlorophyll (mg/g)	Carotenoid (mg/g)
FRC	0.709 ± 0.008 a	0.216 ± 0.002 a	3.281 ± 0.011	0.926 ± 0.010 a	0.171 ± 0.002 a
FR5	0.614 ± 0.012 b	0.185 ± 0.003 c	3.311 ± 0.016	0.800 ± 0.015 c	0.149 ± 0.003 b
FR15	0.575 ± 0.014 c	0.176 ± 0.006 d	3.264 ± 0.026	0.751 ± 0.020 d	0.140 ± 0.004 c
FR30	0.558 ± 0.020 c	0.174 ± 0.004 d	3.206 ± 0.038	0.731 ± 0.024 d	0.129 ± 0.005 d
FR45	0.638 ± 0.010 b	0.196 ± 0.002 b	3.258 ± 0.011	0.834 ± 0.012 b	0.152 ± 0.002 b
	*p* < 0.001	*p* < 0.001	*p* = 0.078	*p* < 0.001	*p* < 0.001

The values are the mean ± SEM of three replicates, and the letters indicate the significant differences among different treatments (*p* < 0.05); significant results of one-way ANOVA are shown.

## Data Availability

The original contributions presented in this study are included in the article/Supplementary Materials; further inquiries can be directed to the corresponding authors.

## Supplementary Materials

The following supporting information can be downloaded at: https://www.mdpi.com/article/10.3390/plants14010139/s1, Figure S1: Spectral values of treatments, red LEDs peak at 660 nm, far-red LEDs peak at 730 nm; Figure S2: Morphology of lettuce under different treatments; Figure S3: Identification of the highly correlated gene modules in WGCNA; Figure S4: Dynamic expression of three phytochrome-responsive genes under FRC treatment; Figure S5: Dynamic expression of three phytochrome genes under FRC treatment; Figure S6: Dynamic expression of five hormone metabolism or transport-related genes under FRC treatment; Table S1: RT-qPCR primer sequences; Table S2: Dynamics of lettuce morphology at different days after treatment (DAT) harvests; Table S3: Shoot dry matter content (DMC) and root development of lettuce under different treatments; Table S4: Net photosynthetic rate (Pn), stomatal conductance (Gs) and intercellular CO2 concentration (Ci) of lettuce under different treatments; Supplemental Data Set 1. Hormone contents in stem tips; Supplemental Data Set 2. Expression of all significant DEGs; Supplemental Data Set 3. Data of WGCNA analysis.
