# Supplementary material for: Intermittent Supplementation with Far-Red Light Accelerates Leaf and Bud Development and Increases Yield in Lettuce
Source: Plants (Basel). 2025 Jan 6;14(1):139. doi: 10.3390/plants14010139 (PMC11723179; doi:10.3390/plants14010139)
Supplement: Supplementary file 1 [file plants-14-00139-s001.zip › Supplementary Table and Figure.pdf]

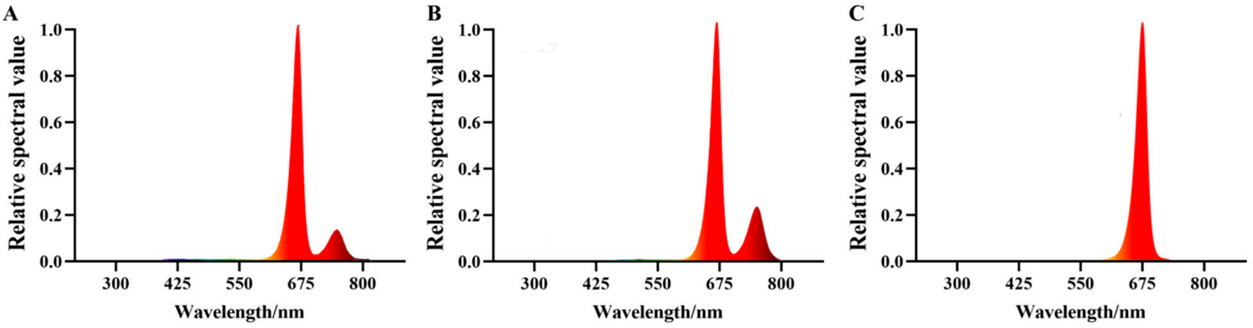

Supplementary Figure 1. Spectral values of treatments, red LEDs peak at 660nm, far-red LEDs peak at 730nm. **(A)** FRC, far-red LEDs constant irradiation. **(B)** FR5/FR15/FR30/FR45, far-red LEDs on. **(C)** FR5/FR15/FR30/FR45, far-red LEDs off.

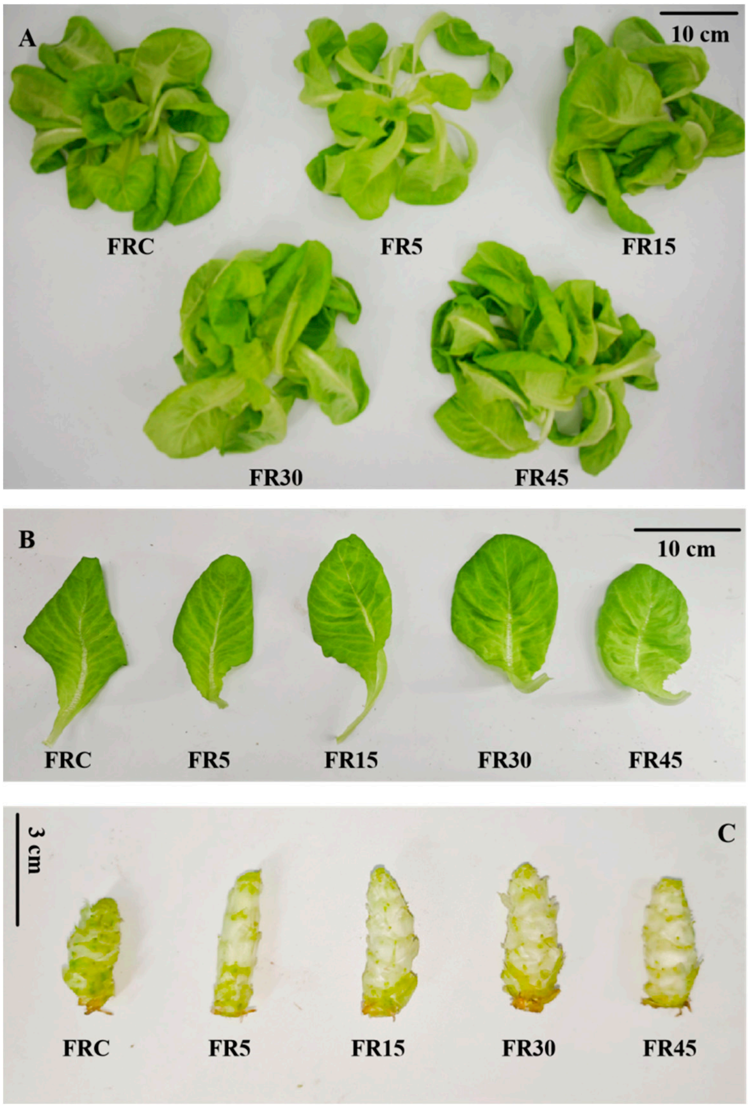

Supplementary Figure 2. Morphology of lettuce under different treatments. Overall morphology **(A)**, leaf morphology **(B)** and stem morphology **(C)** of and lettuce under different treatments.

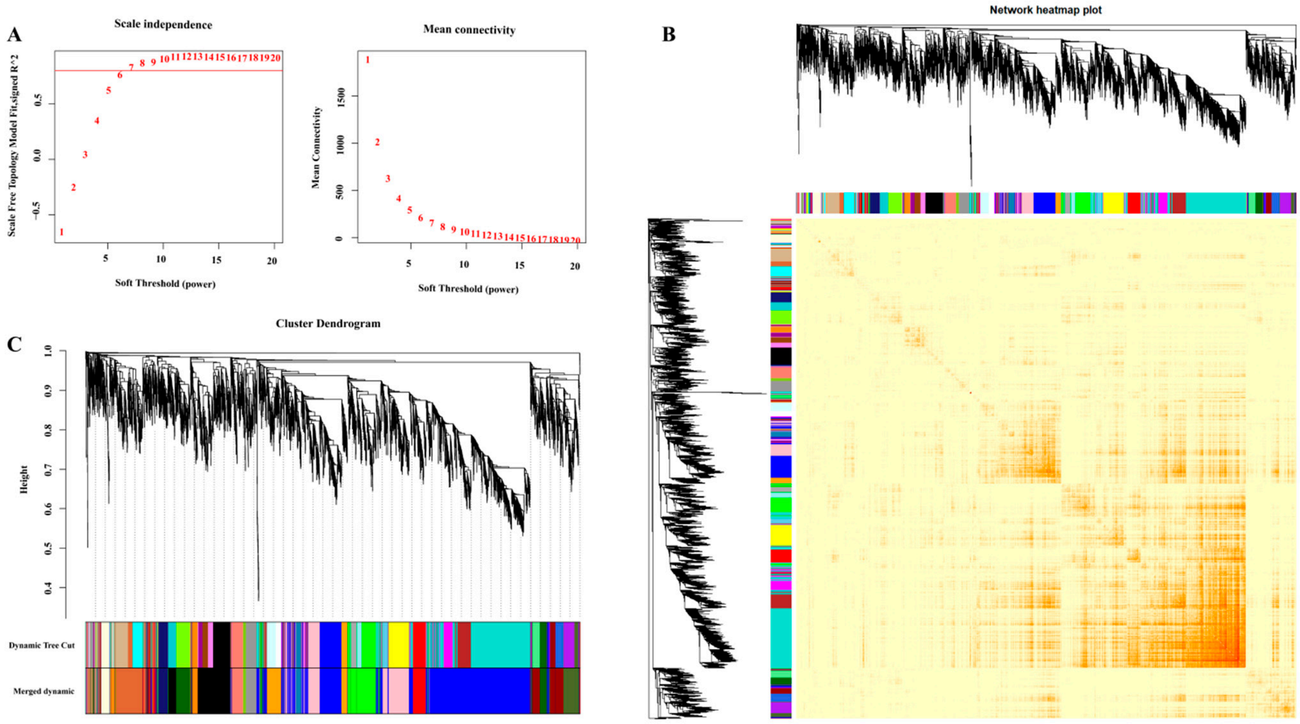

Supplementary Figure 3. Identification of the highly correlated gene modules in WGCNA. **(A)** Determination of the soft thresholding power. The y-axis of the left panel represents the scale-free fit index and the y-axis of the right panel represents the mean connectivity. The x-axis represents power value. **(B)** The gene expression clustering dendrogram and co-expression topology heat map illustrate gene expression similarity, with darker colors indicating higher expression similarity between genes. **(C)** Dendrogram of differentially expressed genes clustered based on the dissimilarity measure (1-TOM). The colors represent the identified gene modules and the merging process.

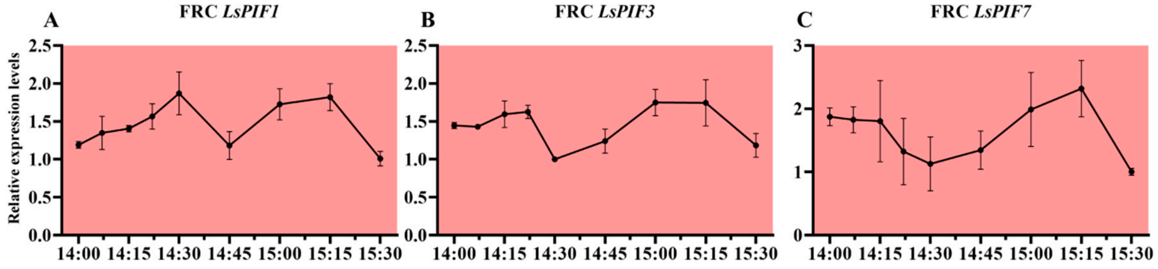

Supplementary Figure 4. Dynamic expression of three phytochrome-responsive genes *LsPIF1* **(A)**, *LsPIF3* **(B)**, and *LsPIF7* **(C)** under FRC treatment. The values are the mean ± SEM of three replicates.

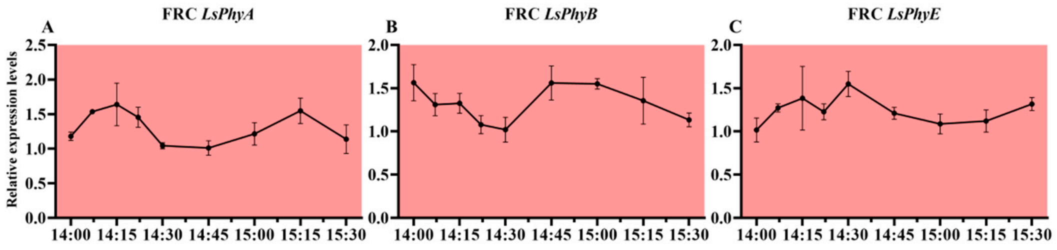

Supplementary Figure 5. Dynamic expression of three phytochrome genes *LsPhyA* **(A)**, *LsPhyB* **(B)**, and *LsPhyE* **(C)** under FRC treatment. The values are the mean ± SEM of three replicates.

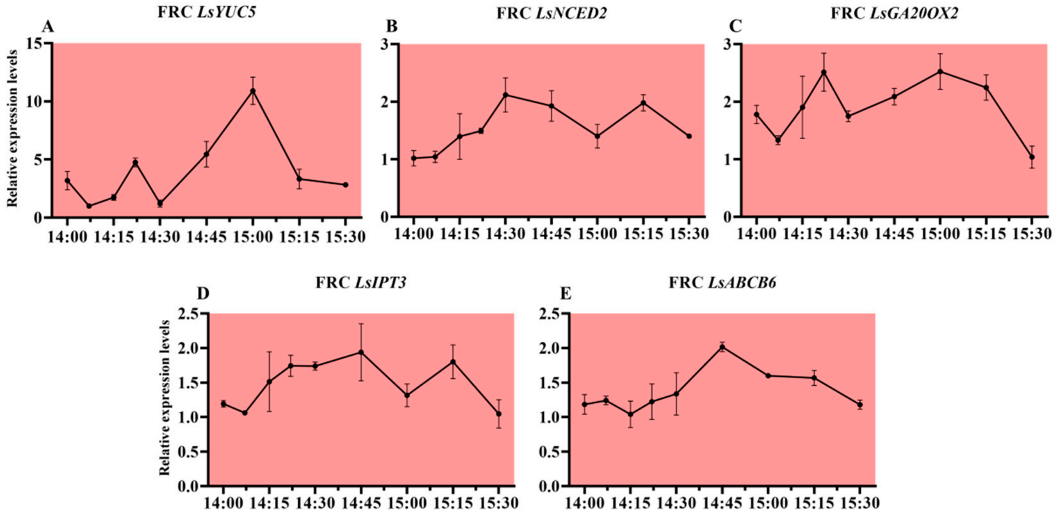

Supplementary Figure 6. Dynamic expression of five hormone metabolism or transport-related genes *LsYUC5* **(A)**, *LsNCED2* **(B)**, *LsGA20OX2* **(C)**, *LsIPT3* **(D)**, and *LsABCB6* **(E)** under FRC treatment. The values are the mean ± SEM of three replicates.

Supplementary Table 1. RT-qPCR primer sequences

Gene	Primer Sequence（F）	Primer Sequence（R）
Ls18S	GTGAGTGAAGAAGGGCAATG	CACTTTCAACCCGATTCACC
LsActin	AGGGCAGTGTTTCCTAGTATTGTTG	CTCTTTTGGATTGTGCCTCATCT
LsPIF1	ACCGCTTCCTCAGCAGAATC	GATCTTTGCTGCTGCTTGGC
LsPIF3	CCAATGCCGGGGTTTCCTAT	TGTTTGCAACATGCGACTGA
LsPIF7	GGTCCGACCTTTGTTTATCCTA	GTAGTCGCTAAGCCTGGAAA
LsPhyA	CTTCACACTAGTGCTTTGTGTC	ACTTGACTTATACATGCACCCA
LsPhyB	CCATGCTCAATACATGGCTAAC	TAACGTAATGGAAACGGAATGC
LsPhyE	CCTCAAAGATGAAGACACGTTG	ATCTTCACCAAAAGACACTTGC
LsGA20OX2	ACCACCACGAAGTCCTCCCATC	CGGTGATGACATCGGTGTTCTGG
LsNCED2	CTGTTTTCCCGAAAGCAATAG	TCATACCGTCCAACCGTCTC
LsYUC5	CCGTCGAGTTCGTTAATGGC	TTAGCAAAGAACTCGGTTTCCTGA
LsIPT3	CCAAGTTGACGGATGACGGA	TGTGCAGTACAAGGCCACAA
LsLOG5	GAATTCATGACAAGCCCGTGG	AGACAATGATGTGGCGCTGA
LsABCB6	CCCACGATGAACTAATCGCC	TCTCACTGGCATCCTTCTTGG
LsGH3.6	TTCATCGCAACAATGGCGTG	CGATACCTGTAAAGACCTGCGT
LsSAUR71	CGGCTACGAACAAAAAGGCG	CTTCGAGGTTGTCAGACGCT

Supplementary Table 2. Dynamics of lettuce morphology at different days after treatment (DAT) harvests.

Treatments	Shoot weight (g/plant)				Stem length(cm)				No. of leaves and buds			
	7 DAT	14 DAT	21 DAT	28 DAT	7 DAT	14 DAT	21 DAT	28 DAT	7 DAT	14 DAT	21 DAT	28 DAT
FRC	19.29±0.66 c	39.81±0.76 b	79.93±2.32 bc	110.61±2.70 ab	1.531±0.052 b	2.026±0.023 b	3.218±0.034 b	4.794±0.141 c	23.39±0.39 b	31.33±0.26 b	42.17±0.41 b	49.75±0.50 ab
FR5	19.41±0.68 c	42.21±1.13 ab	76.97±2.26 c	106.48±3.42 b	1.779±0.078 a	2.286±0.023 a	3.929±0.076 a	6.113±0.348 a	23.44±0.29 b	32.14±0.46 ab	39.33±0.40 c	48.63±0.85 b
FR15	21.48±0.70 b	41.98±1.04 ab	85.03±2.79 ab	111.09±2.01 ab	1.806±0.089 a	2.325±0.034 a	3.817±0.046 a	4.994±0.187 bc	24.33±0.37 a	32.63±0.29 a	42.50±0.35 b	50.00±0.72 ab
FR30	23.73±1.19 a	44.10±1.43 a	89.29±2.64 a	115.66±2.54 a	1.803±0.084 a	2.311±0.027 a	3.958±0.092 a	5.363±0.190 b	24.50±0.45 a	33.00±0.46 a	44.83±0.72 a	51.25±0.67 a
FR45	20.91±0.69 b	44.06±1.80 a	85.10±3.27 ab	115.41±2.38 a	1.724±0.056 a	2.339±0.037 a	3.908±0.096 a	4.475±0.071 d	24.41±0.39 a	32.56±0.55 a	42.58±0.53 b	51.63±0.75 a
p	< 0.001	0.116	0.015	0.092	0.048	< 0.001	< 0.001	< 0.001	0.058	0.017	< 0.001	0.026

The values are the mean ± SEM of three replicates,and the letters indicate the

significant differences among different treatments ($p < 0.05$), significant results of one-way ANOVA are shown.

Supplementary Table 3. Shoot dry matter content (DMC) and root development of lettuce under different treatments.

Treatments	DMC (%)	Root length(cm)	Root weight(g)	Root mass fraction (%)
FRC	2.172±0.044 ab	23.25±0.54 ab	3.100±0.180	3.978±0.093 ab
FR5	2.053±0.046 b	21.00±0.85 b	2.767±0.164	3.836±0.102 abc
FR15	2.233±0.082 ab	22.41±0.60 ab	2.750±0.086	3.652±0.046 c
FR30	2.312±0.021 a	24.50±0.72 a	3.067±0.117	3.775±0.046 bc
FR45	2.302±0.028 a	21.75±0.97 ab	3.100±0.063	4.070±0.037 a
p	0.002	0.016	0.116	< 0.001

The values are the mean ± SEM of three replicates,and the letters indicate the significant differences among different treatments ($p < 0.05$), significant results of one-way ANOVA are shown.

Supplementary Table 4. Net photosynthetic rate (Pn), stomatal conductance (Gs) and intercellular CO_2 concentration (Ci) of lettuce under different treatments.

Treatments	Pn (μmol/m²/s)			Gs (mol/m²/s)			Ci(μmol/mol)		
	Far-red lights on	Far-red lights off	Mean	Far-red lights on	Far-red lights off	Mean	Far-red lights on	Far-red lights off	Mean
FRC			5.371±0.267			0.096±0.011 c			292.02±8.56 b
FR5	5.277±0.091 b	5.699±0.271 a	5.488±0.151	0.179±0.014 a	0.149±0.013	0.164±0.010 a	336.72±3.44 a	322.75±3.59 ab	329.73±3.17 a
FR15	5.719±0.180 ab	5.112±0.383 ab	5.415±0.222	0.136±0.009 b	0.159±0.032	0.148±0.016 ab	316.91±4.05 ab	325.54±10.57 ab	321.22±5.55 a
FR30	5.507±0.278 ab	4.665±0.326 b	5.086±0.241	0.133±0.009 b	0.148±0.018	0.141±0.010 ab	318.29±4.64 ab	332.12±8.35 a	325.20±5.01 a
FR45	5.831±0.197 a	4.728±0.192 b	5.280±0.212	0.143±0.030 ab	0.116±0.027	0.129±0.020 b	298.48±23.41 b	305.32±16.44 b	301.90±13.68 b
p	0.002	< 0.001	0.125	0.005	0.111	< 0.001	0.002	0.013	< 0.001

The values are the mean ± SEM of three replicates,and the letters indicate the significant differences among different treatments ($p < 0.05$), significant results of one-way ANOVA are shown.
